# A systematic review of interventions to promote physical activity in six Gulf countries

**DOI:** 10.1371/journal.pone.0259058

**Published:** 2021-10-28

**Authors:** Elizabeth A. Nash, Julia A. Critchley, Fiona Pearson, Susanne F. Awad, Laith J. Abu-Raddad, Farah M. Abu-Hijleh, Peijue Huangfu

**Affiliations:** 1 MBBS Student, St George’s, University of London, London, United Kingdom; 2 Population Health Research Institute, St George’s, University of London, London, United Kingdom; 3 Evidence Synthesis Group, Population Health Sciences Institute, Newcastle University, Newcastle upon Tyne, United Kingdom; 4 Infectious Diseases Epidemiology Group, Weill Cornell Medicine–Qatar, Cornell University, Doha, Qatar; 5 World Health Organization Collaborating Centre for Disease Epidemiology Analytics on HIV/AIDS, Sexually Transmitted Infections, and Viral Hepatitis, Weill Cornell Medicine–Qatar, Doha, Qatar; 6 Department of Population Health Sciences, Weill Cornell Medicine, Cornell University, New York, New York, United States of America; 7 Department of Public Health, College of Health Sciences, QU Health, Qatar University, Doha, Qatar; 8 Department of Public Health, College of Health Sciences, Academic Quality Affairs Office, QU Health, Qatar University, Doha, Qatar; Universidad Miguel Hernandez de Elche, SPAIN

## Abstract

Physical activity (PA) levels are low in Gulf Cooperation Council countries (GCC; Bahrain, Kuwait, Oman, Qatar, Saudi Arabia, United Arab Emirates). We carried out a systematic review (PROSPERO registration number 131817) to assess the effect of interventions to increase PA levels in this region. We also assessed their effects on anthropometry and cardiovascular risk. A systematic search of six databases (Medline, EMBASE, SPORTDiscus, CINAHL, Cochrane, Web of Science) was performed to identify randomized and non-randomized intervention studies performed in adults and children published between January 1985 and November 2020. We included studies published in English or Arabic, and included PA interventions regardless of setting, delivery, and duration. The primary outcomes were changes in PA duration and intensity. Secondary outcomes included anthropometric measures (e.g., weight, body mass index) and cardiovascular risk profiles (e.g., lipid measures, blood glucose). Two independent reviewers screened studies in accordance with pre-determined criteria, extracted data, assessed risk of bias (Cochrane Risk of Bias 2 and Newcastle Ottawa Scale) and undertook a narrative synthesis. From 13,026 records identified, 14 studies were included. Nine studies focussed exclusively on changing PA behaviour, resulting in statistically significant increases in step count ranging from an additional 757 steps/day (95% confidence interval [CI] 0–1,513) to 3,853 steps/day (95% CI 3,703–4,002). Five identified studies were multi-component lifestyle interventions, targeting people at higher risk (due to obesity or type 2 diabetes). Evidence for increases in PA from multi-component studies was limited, although improvements were seen in outcomes e.g. body weight and blood lipid levels. In conclusion, relatively few studies have focussed on changing PA behaviour, despite the urgent need in the GCC. Limited evidence suggested that pedometer-based programmes encouraging step counting and walking were effective in promoting PA, at least in the short term. Policies to roll out such interventions should be implemented and evaluated.

## Introduction

The Gulf Cooperation Council (GCC) countries (Bahrain, Kuwait, Oman, Qatar, Saudi Arabia and United Arab Emirates (UAE)) in the Arabian Peninsula experience some of the highest rates of diabetes and obesity in the world. Over 25% of adults are already living with diabetes [1] and this is projected to increase to over one-quarter of adults by the year 2050 [1]. Disease burden is likely to rise substantially as these relatively young populations age over the coming decades [2–4]. Whilst the explanations for this are clearly multi-factorial, it is also well established that levels of physical activity (PA) are low in this region. A recent systematic review identifying levels of PA and sedentary behaviour research conducted in the GCC countries identified that around 39.0%-42.1% of men and 26.3%-28.4% of women met the internationally recommended PA levels [5]. This is well below the percentage meeting internationally recommended PA levels globally of 76.6% for men and 68.3% for women [6].

There are likely many regional barriers to higher levels of PA, and these may differ somewhat from barriers elsewhere. Environmental factors may be one important barrier; GCC countries are highly urbanised, leading to a transport infrastructure and culture that promotes increased dependence on motor vehicles. This results in limited pavement space, and green or non-developed space which can promote PA [7]. Summer in the GCC is harsh with typical day time temperatures above 40 degrees Celsius, meaning that outdoor exercise for much of the day is potentially unsafe [8]. Gender roles help explain the very low PA levels reported among women; their typical role has been within the home but an influx of migrant domestic workers has reduced the daily level of household chores [9]. Cultural expectations may also prevent women from attending mixed-sex facilities such as gymnasiums, and in some countries a male chaperone is needed for women to take part in organised PA, limiting their accessibility [9]. Historically, PA appears to have been given low priority in society resulting in a lack of facilities, relative absence of peer support, and low parental priority placed upon childhood PA [9,10].

Clearly, interventions to promote PA are of high importance to this region but it cannot be assumed that intervention studies from temperate western countries are generalizable to this very different geographical and cultural environment. A previous literature review on PA in the GCC countries was carried out five years ago, but focussed on the levels of and barriers to PA [5], as very few intervention studies had been carried out at that time. Another recent review identified PA intervention studies in the Arab World [11]. However, no risk of bias assessment was completed hindering interpretation of findings. In addition, authors of this review also did not provide a narrative summary, focussing on tabulating findings. Their objective was to describe the trajectory and quantity of research rather than assess the effects of interventions on changes in PA level as an outcome [11]. Furthermore, the Arab region includes a more heterogeneous population than just the GCC countries [11].

Our key objective was to assess the effect of interventions used to promote PA in both predominantly healthy adults and children in the GCC countries. We aimed to identify and describe the types of intervention that have been tested, the methodological robustness of the studies undertaken, the outcomes of the interventions and where possible whether the patterns of results were in line with those performed in other settings/regions. Secondary aims included reporting any changes in anthropometric and metabolic risk markers. The results of this review can aid future PA research in this region as well as guide the implementation of immediate policy relevant actions.

## Methods

We conducted a systematic review to assess the interventions promoting PA in the GCC countries. Our methods have previously been published as a protocol [12] (PROSPERO registration number 131817) and are summarised here.

### Inclusion and exclusion criteria

We included both randomized control trials (RCTs) and quasi-experimental studies (cohort studies) which report the difference in PA pre- and post-intervention, studies with a comparator group, interrupted time series studies and propensity score matching studies. All studies that aimed to promote PA amongst generally healthy children (over 5 years old) and adults in GCC countries were eligible, regardless of ethnicity. Studies among specific patient populations such as those with diabetes were included but studies of exercise rehabilitation (e.g., after surgery or myocardial infarction) were excluded. We included interventions regardless of setting (e.g., community, home-based, primary care), delivery mode (e.g., face-to-face, self-motivated), and intervention period and intensity. PA interventions could be either standalone or as part of a multi-component approach to health including advice on diet, smoking cessation, and management of cardiovascular risk factors. We only included such multi-component programmes if they also reported on changes in PA using a recognised self-report or objective measure. Any type of PA programme was included such as online or face-to-face, counselling, use of PA trackers such as pedometers, or group exercise. Control groups included those with no intervention, a less intense or minimal intervention (such as brief, one-off advice to increase exercise). We thus excluded studies with only one-time short consultations at the beginning of the intervention.

Our primary outcome was a change in PA level (i.e., duration and intensity), measured either through recognised self-report questionnaires or more objectively (e.g., using pedometers, smart phones or accelerometers). Apart from the changes in PA levels, several studies also reported changes in sedentary behaviours (e.g., sitting time/day), we therefore recorded them as important secondary outcomes. Other outcomes of interest were anthropometry or changes in other cardiovascular risk factors such as blood lipids or blood pressure (BP).

### Search strategy

We searched six databases, including Medline (via Pubmed), Embase (via Ovid), SPORTDiscus (via EBSCOhost), CINAHL (via EBSCOhost), Web of Science, and Cochrane library, for published studies and review articles from 1^st^ January 1985 until 21^st^ November 2020. We used Medical Subject Heading (MeSH) terms (such as exercise, training, sports and fitness) and keywords (including but not limited to run*, cycl* and swim*) to create a highly sensitive search strategy (S1 Appendix). We also performed a citation search for relevant reviews in this area, and searched in grey literature including conference abstracts and meeting proceedings. We included published studies in either English or Arabic. Authors were contacted in the absence of full-text papers or critical information in articles.

### Selection of studies

Screening of studies was carried out using Rayyan [13]. Titles and abstracts of records retrieved from searches were screened for inclusion by two of three researchers independently (EN, PH, JAC) and any differences in agreement were resolved by discussion. Data extraction for key characteristics and outcomes was carried out in excel after piloting a specifically designed form, and performed by EN and PH. We also contacted five authors where important data (particularly the season of the intervention delivery, or ethnicity of included participants) was missing from the publications and received three responses.

### Risk of bias assessment

RCTs were assessed using the Cochrane risk of bias tool [14] following relevant signalling questions to assess i) bias arising from the randomization process, ii) bias due to deviation from intended interventions, iii) bias due to missing outcome data, iv) bias in measurement of the outcome, and v) bias in selection of the reported results. Bias arising from the randomisation process was judged low if both a method of randomisation and concealing allocation was clearly described, along with no clear obvious baseline differences between groups. If not enough information was provided, the study was judged to have some concerns. Bias due to deviation from intended interventions was judged low if study participants did not change between groups. Bias due to missing outcome data was judged low if less than 20% of participants were lost to follow-up. Bias due to measurement outcome was judged low if an objective measure was used for the main results (e.g. pedometers). Bias due to selective reporting of outcomes was judged low if outcomes were pre-specified. Bias due to incomplete reporting was judged low if measurement methods, methods of analysis and outcomes were specified in advance. Bias due to sample size calculations was judged low if this was performed in advance and attained in the study. Non-randomized intervention (NRI) studies were assessed using the Newcastle-Ottawa Scale (NOS) [15] on aspects of study selection, comparability, and outcome assessment. These tools were used independently by researchers (EN and PH) with disagreements resolved by a third researcher (JAC).

### Narrative synthesis

Due to the heterogeneity of studies identified (i.e., intervention, outcomes, and study population), we narratively assessed the studies by their intervention design i.e., PA intervention only versus PA involved in a multicomponent programme. Several studies reported “step count/day” as an outcome, therefore we narratively synthesised these studies in a forest plot without calculating an overall estimate, and compared the individual study results with a recent global systematic review [16].

## Results

**Fig 1:**
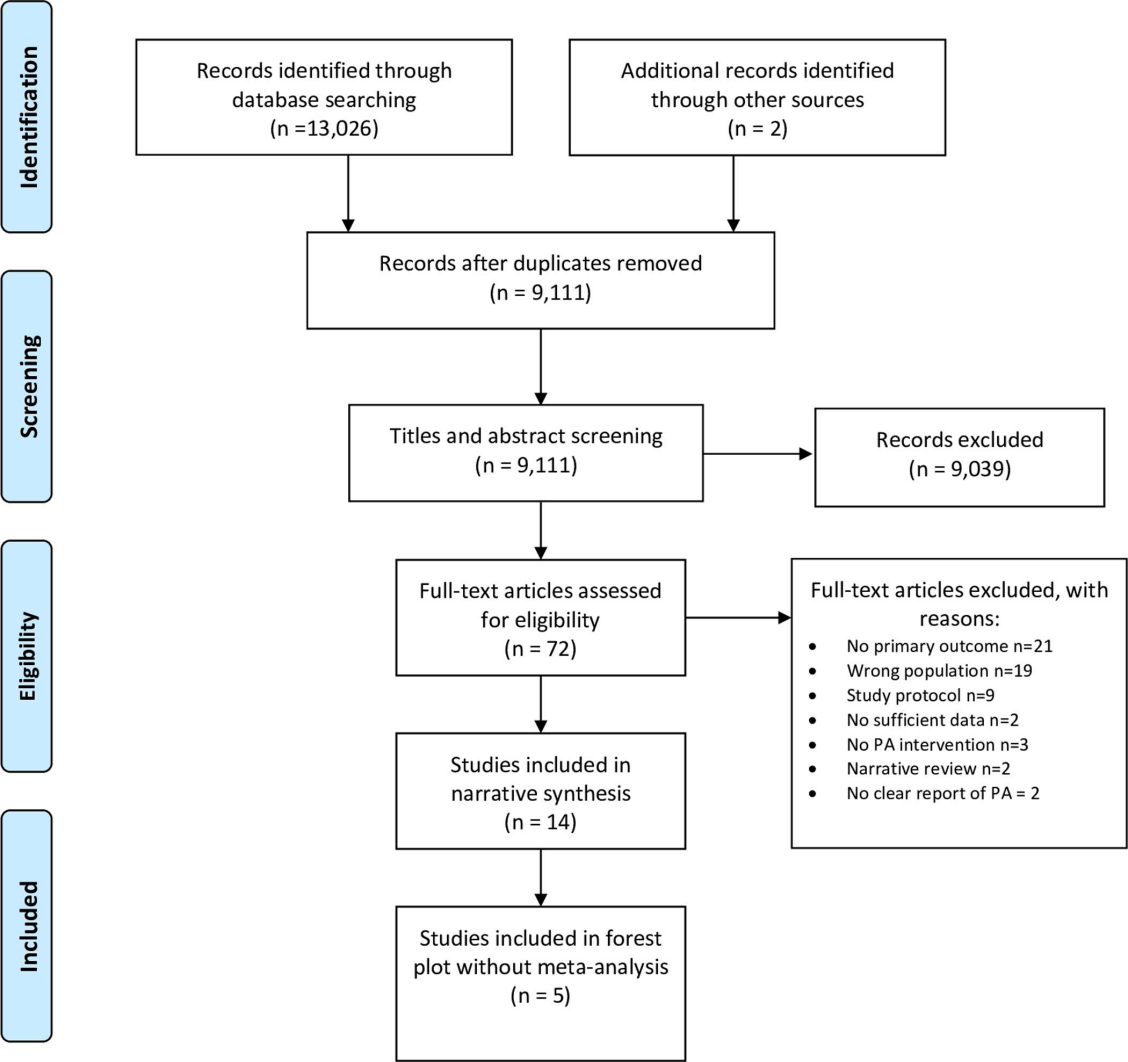
PRISMA flow chart for study selection.

Bibliographic database searching in November 2020 returned 13,026 results, with two further articles identified from communication with one study author (i.e., unpublished results). Once de-duplication had been performed in Endnote, 9,111 results remained for title and abstract screening (Fig 1). Seventy-two of these papers were included for full-text screening based on our inclusion criteria. Among these, we excluded 21 studies reporting no primary outcomes of interest (i.e., PA measurement), 19 including different populations (i.e., non-GCC populations or pregnant women), 9 study protocols, 3 studies that involved no specific PA intervention, 2 studies with insufficient information despite contacting the authors, 2 narrative reviews and 2 not clearly reporting measures of PA change (S2 Appendix). Thus 14 studies remained after full-text screening and have been included in this systematic review.

### Study characteristics

Table 1 presents the study characteristics of the included studies. Of the fourteen included studies, eight were RCTs [17–24], two of which were clustered [23,24], and six were NRI studies [25–30]. Seven of the studies were conducted in Qatar [22,25–30], three in Saudi Arabia [18,21,24], two in Oman [17,23], and one study each in Kuwait [19] and UAE [20]. Most of the interventions took place during the cooler autumn or winter months, though some appear to have continued all year around. The sample sizes ranged from 39 [18] to 15,947 [26]. Eight studies included both male and female participants, four studies with only females [17,20,21,29], and two only males [18,24].

**Table 1:** Characteristics of the included studies.

Author, Year	Study design	Country	Setting	Age (mean ± SD)	Sample size (% female)	PA Intervention	Multicomponent intervention	Duration of intervention	Follow up period	Outcomes measured
Al-Anqodi 2018 [17]	RCT	Oman	Community	Not reported	42 (100)	Smartphone app (step-count pedometer)	3-day food diary at baseline and end	5 weeks	None	Mean active time, mean step count
Alduhishy 2012 [18]	RCT	Saudi Arabia	Community	Not reported	39 (0)	Active group: instructed to walk 10,000+ steps per day, ≥5 days per week for 12 weeks	None	12 weeks	None	Steps/day, glucose, fasting insulin, body mass
Al-Kuwari 2015^i^ [27]	NRI	Qatar	Community (“Step into Health”)	Not reported	103 (38.4)	Pedometer and goal setting to reach and maintain 10,000 steps per day	None	12 months	None	Steps/day
Al-Kuwari 2016^i^ [25]	NRI	Qatar	Community (“Step into Health”)	41.3±10.7	970 (27.3)	Pedometer and goal setting to reach and maintain 10,000 steps per day	None	3 months	None	Steps/day
Al-Kuwari 2017^i^ [30]	NRI	Qatar	Community (“Step into Health”)	Not reported	268 (Not reported)	Pedometer and goal setting to reach and maintain 10,000 steps per day	None	12 months	None	Steps/day
Alghafri 2018 [23]	Cluster RCT	Oman	Primary- Care	44.2±8.1	232 (59.1)	Face-to-face 20min consultations at 0, 4, 8 weeks and monthly messages to encourage PA participation. Given pedometers and advice on weight management	Dietary intervention with face-to-face consultations (20mins at 0, 4, 8) weeks	12 months	None	Steps/day, sitting time, weight, BMI, systolic and diastolic BP, HbA1c, lipids (total cholesterol, HDL, LDL, triglycerides)
Allafi 2020 [19]	RCT	Kuwait	School	Not reported	225 (51.1)	Given advice on pedometers and incentive of 10 stickers if 3000 steps performed. Measurements taken during 5x50 minute exercise sessions	None	Not reported	None	Steps/day
Al-Mohannadi 2019 [28]	NRI	Qatar	Workplace (“Step into Health”)	Not reported	54 (43.4)	Workplace challenge was promoted through internal announcements; pedometer provided	Health tips through automated emails and SMS. Incentives for participants averaging 10,000 steps/day after 3-month. Weekly ranking system internally.	4 months	5 months	Steps/day
Kutbi 2019 [24]	Cluster RCT	Saudi Arabia	School	14.45 ± 2.32	148 (0)	60-minute session in health education in 1^st^ and 5^th^ week	Health education, group counselling, group discussion on healthy lifestyle	2 months	None	PA frequencies (e.g. walking, running, cycling, days/week, how many minutes) time spent in sedentary behaviours
Platat 2010 [20]	RCT	UAE	University	Not reported	42 (100)	10 week pedometer programme with individual daily step goal of baseline step count + 3000 steps	None	10 weeks	None	Steps/day, anthropometry, blood pressure, biochemical parameters (blood glucose, triglycerides, HDL, LDL, total cholesterol, insulin)
Quronfulah 2019 [21]	RCT	Saudi Arabia	Workplace	43.5±11.1	66 (100)	Weekly text messages and computer prompts at workplace as reminders to break up sitting time	None	12 weeks	None	Sedentary behaviour (sitting time), light-intensity PA
Sayegh 2016^i^ [29]	NRI	Qatar	Community (“Step into Health”)	37.4±11.7	549 (100)	Pedometer goal setting to reach and maintain 10,000 steps/day	None	6 months	6 months	Steps/day
Taheri 2020 [22]	RCT	Qatar	Primary- Care and community	42.1±5.6	158 (27.0)	PA support focusing on walking (aim for 10,000+ steps per day), & increase unsupervised activity to 150+ mins per week. Directed to smartphone apps	Dietary intervention, medications stopped and reintroduced if necessary	12 months	None	Increase in PA, weight, waist-circumference, HbA1c, sitting time, lipid lowering medication use, blood pressure, lipids, insulin sensitivity, anxiety and depression
Walt 2016^i^ [26]	NRI	Qatar	Community (“Step into Health”)	Not reported	15,947 (46.4)	Pedometer given and asked to reach 10,000 steps per day; reminders by regular emails and texts	None	36 months	None	Steps/day

^i^Studies were based on samples taken from the same community prevention study as part of ‘Step into Health Project’. There is likely to be overlap between participants, the extent to which could not be determined.

Two studies were conducted in a primary care setting [22,23]; two were carried out among school children [19,24]; two were targeted at workplaces [21,28]; ]; one study was conducted in university students [20]; and the remaining studies took place in community settings. The majority of studies focussed on generally healthy adults, while three studies that specifically recruited patients who were overweight or had type 2 diabetes (T2DM) [18,22,23].

Primary care interventions included regular face-to-face consultations to increase PA uptake along with monthly motivational messages [22]. School-based interventions included the provision of a pedometer as well as a rewards-based system to promote increased step count during exercise sessions [19]; other methods used health education alongside group counselling sessions to promote PA uptake [24]. Workplace PA interventions included reminders for employees to break up sitting time [21], and the promotion of workplace step count challenges [28]; with one of the studies performed as part of the ‘Step into Health’ project in Qatar [28]. The university-based intervention designed step count goals whilst implementing the use of pedometers to obtain targets [20]. All of the community-based interventions included goal-setting of 10,000 steps per day, implementing the use of a pedometer or a pedometer app on smartphones; among them five were part of the ‘Step into Health’ programme [25–27,29,30].

Intensity of interventions ranged from the simple provision of body trackers alongside PA advice to more complex multi-faceted programmes. Interventions such as encouragement to measure step count using pedometers were used together with weekly reminders to upload PA data. More intensive interventions included programmes involving frequent face-to-face consultations or exercise classes, along with accelerometers or pedometers and monthly motivational messages to encourage PA participation. The control groups received either normal care, a shorter or less intensive intervention, or a different intervention altogether. Five of the fourteen studies also included PA interventions as part of a multi-component programme with the majority also focussing on dietary changes and health education [17,22–24,28].

Apart from the primary outcomes of interest reported, four studies [18,20,22,23] also reported weight-related outcomes (i.e., body mass index [BMI], waist circumference), along with other obesity-related measures (e.g., BP, fasting blood glucose/haemoglobin A1c [HbA1c], blood lipids). Most studies measured PA using an objective instrument i.e., pedometers. Two studies [21,23] used both objective and subjective measures of PA (respectively pedometers/accelerometers and recognised, validated questionnaires for PA such as the International Physical Activity Questionnaire [IPAQ] [31,32] or Global Physical Activity Questionnaire [GPAQ] [33,34]).

The intervention period varied between studies with a minimum of 5 weeks to 36 months. Two of the fourteen studies also included a period of follow up of 5 [28] and 6 months [29] respectively.

### Risk of bias assessment

**Fig 2:**
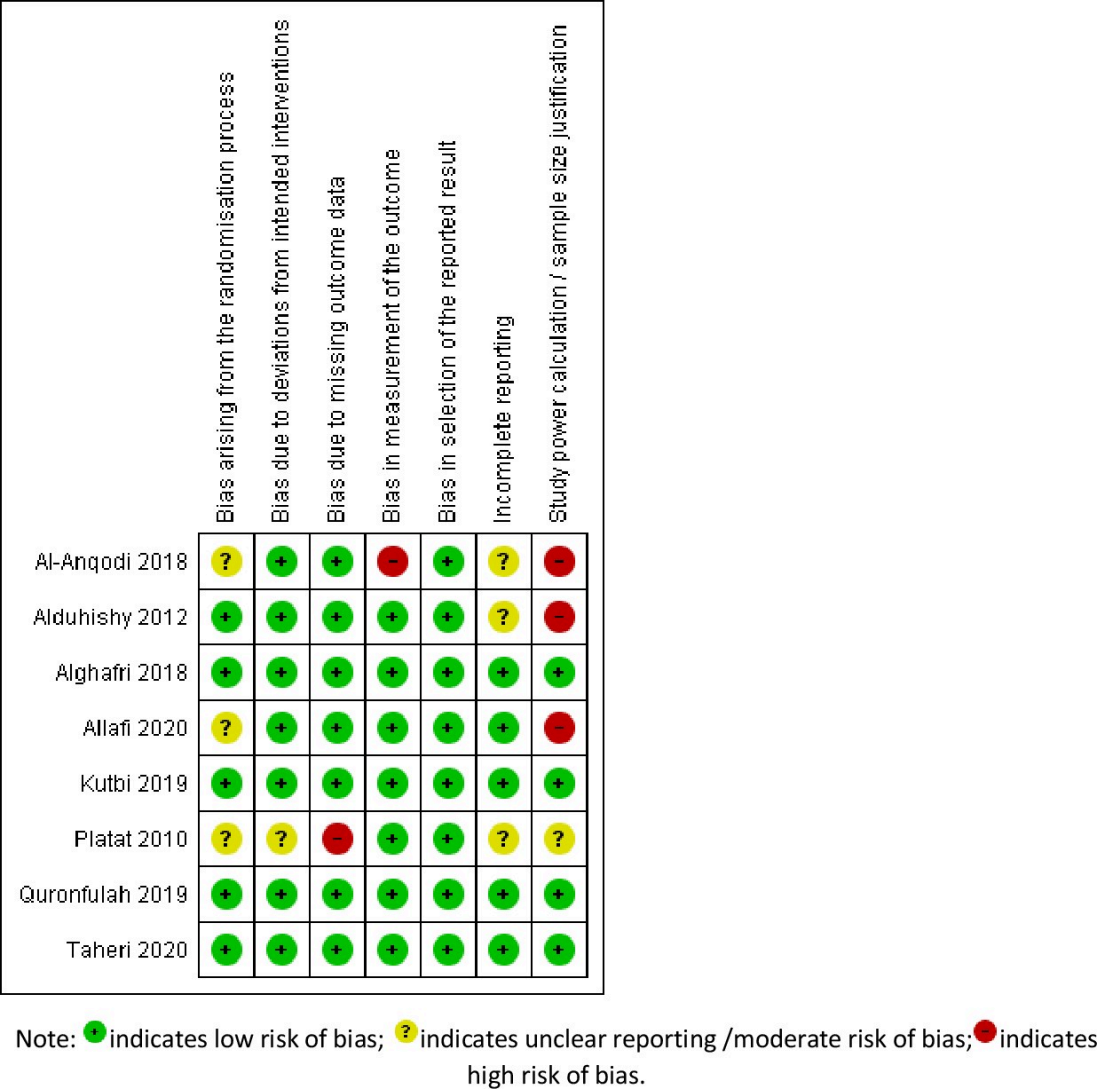
Risk of bias summary of included randomized control studies.

Fig 2 illustrates the risk of bias summary of included RCTs. The signalling question “bias arising from the randomization process” was most often identified, in part because studies did not clearly report how the allocation sequence was concealed rather than because the randomization process itself allowed bias. Other bias exists amongst individual studies due to the measurement of the outcome (self-reported PA outcomes being subjective and at risk of bias). One study [17] used two different methods to measure outcomes of interest: in the intervention, a smartphone step count pedometer was used, while in the control group, a PA log was requested. The lack of information reported in studies among certain domains might have disguised underlying biases that could not be identified. Apart from the biases identified by the Cochrane risk of bias tool, we also found some studies had issues with incomplete reporting [17,18], that is under-reporting the actual intervention effect (i.e., between intervention and control group) while emphasizing the within group change. Moreover, a few studies failed to explain their sample size estimation [17–19].

Table 2 shows the NOS risk of bias assessments undertaken for NRI studies. The main biases identified were lack of representativeness of the exposed cohort and comparability of cohorts. All studies selected the participants on a volunteering basis rather than systematic random selection. None of them considered any adjustment for potential confounders (e.g. seasonal effect, socioeconomic status).

**Table 2:** Risk of bias summary for NRI studies using Newcastle Ottawa Scale.

Author year	Selection				Comparability of cohorts on the basis of the design or analysis	Outcome			Final score
	Representativeness of the exposed cohort	Selection of the non-exposed cohort	Ascertainment of exposure	Demonstration that outcome of interest was not present at start of study		Assessment of outcome	Was follow-up long enough for outcomes to occur	Adequacy of follow up of cohorts	
Al-Kuwari, 2016^ii^ [25]	0	1	1	1	0	1	1	1	6
Al-Mohannadi 2019 [28]	0	1	1	1	0	1	1	1	6

^i^Based on Newcastle Ottawa Scale (NOS). The maximum score of NOS is 9. Selection domain has a maximum score of 4, each subdomain ranges from 0–1; comparability domain has a maximum score of 2 (range 0–2); outcome domain has maximum score of 3, each subdomain ranges from 0–1.

^ii^Al-Kuwari 2015 [27], Al-Kuwari 2017 [30], Sayegh 2016 [29], and Walt 2016 [26] are of the same study design as Al-Kuwari 2016 [25] from the “Step into Health” programme (except variate intervention period and study sample, see details in Table 1); thus only Al-Kuwari 2016 [25] was presented in this table.

#### Effects of interventions on PA outcomes

The 14 included studies were heterogeneous in terms of their design, focus and population (see Tables 1 and 3). Five of those included were multi-component lifestyle interventions, focussing on other lifestyle changes (particularly dietary behaviours) as well as PA.

**Table 3:** Table of results for included studies.

Author, Year	Study design	Country	Study Population	PA Intervention	Multicomponent intervention	PA results	Other results
Al-Anqodi 2018 [17]	RCT	Oman	Healthy Omani female	Smartphone app (step-count pedometer).	Both groups asked to keep a 3-day food diary at baseline and the last week of intervention.	After intervention app group were active for 48.3min/day (SD25.1), compared to 35.89 (SD24.7) for control group (P = 0.13). The app group had significant (within group) increase in step count (P = 0.001) and active time (P = 0.006)	Comparing to the control group, there was a decrease among the app group on energy intake (P = 0.04) and carbohydrate intake (P = 0.009), and increase of protein intake (P = 0.04)
Alduhishy 2012 [18]	RCT	Saudi Arabia	Overweight men with family history of T2DM	Active group: instructed to walk 10,000+ steps per day, ≥5 days per week for 12 weeks	None	Active group increased from 3,781 steps/day (SD 344) to 9,199 (1084) steps/day (P = 0.002); control group increased from 3,298 (SD516) steps/day to 4,863 (SD787) steps/day (P = 0.03). Difference between groups was significant (P<0.05).	Body mass, BMI, and diastolic BP were significantly lower after intervention in active group compared to control group (P<0.05).
Al-Kuwari 2015^i^ [27]	NRI	Qatar	Healthy general adult population enrolled in ‘Step into health project’	Pedometer and goal setting to reach and maintain 10,000 steps per day	None	Largest effect was seen at 3 months (12,376 steps/day) and 4 months (12,321 steps/day) follow up compared to baseline (3,933 steps/day).	Not reported
Al-Kuwari 2016^i^ [25]	NRI	Qatar	Healthy general adult population enrolled in ‘Step into health project’	Pedometer and goal setting to reach and maintain 10,000 steps per day	None	12-week programme showed statistically significant increase in daily step count from 6,833 (SD 4,144) steps at baseline to 10,600 (SD 6,385) steps at week 12.	Not reported
Al-Kuwari 2017^i^ [30]	NRI	Qatar	Healthy general adult population enrolled in ‘Step into health project’	Pedometer and goal setting to reach and maintain 10,000 steps per day	None	Steps increased from 3933±3240 steps/day at baseline to 7507±5416 steps/day at the 12th month (P<0.001)	Not reported
Alghafri 2018 [23]	Cluster RCT	Oman	Adults with T2DM	Face-to-face 20 min consultations at 0,4,8 weeks and monthly messages to encourage PA participation. Given pedometers and advice on weight management	Dietary intervention with face-to-face consultations (20 mins at 0,4,8) weeks	Objectively measured step count in intervention group at 12 months was significantly higher with 757 steps/day more than the control group (P = 0.049). Self-reported PA in intervention group was 246 MET.min/week more at 3 months (P = 0.02), and 447 MET.min/week at 12 months (P = 0.003) compared to the control group. Intervention group had significant fewer sitting hours at 3 months (P<0.001) and 12 months (P<0.001).	Intervention group had significantly lower systolic BP at 3&12 months (−3.8[−6.7 to −0.9]); months (−1.8[−3.5 to −0.1]) and diastolic BP at 12 (−1.6[−2.6 to −0.7].
Allafi 2020 [19]	RCT	Kuwait	School children aged 9–11 attending public schools	Given advice on pedometers and incentive of 10 stickers if 3000 steps performed. Measurements were taken during 5x 50 minute exercise sessions.	None	The feedback and reward group showed significantly higher steps: 3,429 (SD 458), than the feedback group: 2,655 (SD 577) and control group: 2,091 (SD 483) (P<0.01). No significant difference between boys and girls.	Not reported
Al-Mohannadi 2019 [28]	NRI	Qatar	Hospital staff aged 18+ enrolled in ‘Step into health project’	Workplace challenge was promoted through internal announcements; pedometer provided.	Health tips through automated emails and SMS. Participants who averaged 10,000 steps/day received incentives after 3-months. Weekly ranking system internally.	Pre-intervention steps/day at 7,890 (SD 713). Significant increase during the intervention period at 9,270 (SD 672); slight reduction post intervention at 8,998 (SD 683).	Not reported
Kutbi 2019 [24]	Cluster RCT	Saudi Arabia	Male school students between 10–15 years	60-minute session in health education in 1^st^ and 5^th^ week.	Health education, group counselling, group discussion on healthy lifestyle.	No statistically significant differences in total METs between intervention group (2098.41±1922.67) and control group (2216.46 ± 1816.03), P>0.05.	Not reported
Platat 2010 [20]	RCT	UAE	Sedentary, female university students aged 18–35	10 week pedometer programme with individual daily step goal of baseline step count + 3000 steps	None	At baseline mean daily steps were 8146.22 +/- 3457.89 (no significant difference between intervention and control groups). The intervention group significantly increased its daily step count of more than 3000 with no change observed in the control group. The difference was statistically significant (p = 0.02) even after adjustment for confounding variables.	Triglycerides increased in both groups but to a smaller degree in the intervention group compared with the control group.
Quronfulah 2019 [21]	RCT	Saudi Arabia	Male university staff members	Weekly text messages and computer prompts at workplace as reminders to break up sitting time.	None	For objective PA results: intervention group significantly reduced sitting time by 46 min/day (-86.7, -5.2) (difference in difference), P = 0.027; increased light intensity PA by 27.8min/day (-0.5, 56.1), P = 0.054; and increased moderate-to vigorous PA by 16.5min/day (6.9, 56.1) P = 0.001. For self-reported PA results (n = 66): intervention group had 130.9kcal/day (43.6, 218.2) more energy expenditure compared to the control group (P = 0.004)	The intervention group had significant improvement in their social cognitive processes over the intervention period compared to the control group (P<0.001) (both self-efficacy and self-regulation were significantly improved in intervention group).
Sayegh 2016^i^ [29]	NRI	Qatar	Qatari national females aged 18–64	Pedometer goal setting to reach and maintain 10,000 steps/day	None	The median step count at baseline was ~6000 (IQR 3,441 to 10,082) per day which decreased to 5584 (IQR 3,226–9,586) at 6 months but then increased to ~6375 at 12 months.	Not reported
Taheri 2020 [22]	RCT	Qatar	Adults aged 18–50 with T2DM diagnosis within last 3 years and BMI>27	PA support focusing on walking (aim of at least 10,000 steps per day), & increase unsupervised activity to 150+ mins per week. Directed to smartphone apps	Dietary intervention, medications stopped and reintroduced if necessary	Length of time spent sitting per day decreased by 40.8 mins (SD 260.3) in intervention group. MET min/week for walking increased by 151·2 (SD 994·7) in the intervention group but decreased by 235·7 (SD 652·0) in the control group. No significant difference between groups for change in moderate and vigorous PA, and the total MET.min/ week.	Statistically significant reduction in mean bodyweight in the intervention group, 11.98kg, compared with 3.98kg in the control group. Compared with control group, intervention group had a statistically significant improvement in waist-circumference (-11.44, SD 9.9 compared to -4.03, SD 5.68), waist to hip ratio (-0.10, SD 0.08 compared to -0.03, SD 0.05), and fat mass (-9.97, SD 9.06 compared to -2.89, SD 6.41). Reductions in HbA1c (-0.89% SD 1.05 compared to -0.35% SD 1.27), systolic BP (-8.19mmHg SD 12.66 compared to -4.42mmHg SD 11.44), diastolic BP (-5.60mmHg SD 7.34 compared to -2.24mmHg SD 7.88), triglycerides (-0.50mmol/L SD 1.50 compared to -0.13mmol/L SD 0.92) were shown.
Walt 2016^i^ [26]	NRI	Qatar	Healthy general adult population enrolled in ‘Step into health project’	Pedometer given and asked to reach 10,000 steps per day; reminders by regular emails and texts	None	Step count increased from 4830 steps/day in 2013 to 6124 steps/day in 2015. Men performed more steps than women. Participants over 45 years had the highest step count (7010 and 5564 steps/day for men and women respectively).	Not reported

^i^Studies were based on samples taken from the same community prevention study as part of the ‘Step into Health’ project. There is likely to be overlap between participants, the extent to which could not be determined.

#### Interventions focussing only on PA

Nine studies that focussed solely on modifying PA behaviours were identified. Eight of these measured changes in PA only through pedometer step count and hence were focussed primarily on walking and related behaviours. The remaining study also measured sitting time and light-intensity PAs using pedometers [21].

One of these studies was an RCT among Kuwaiti school children [19], randomizing 225 children during five 50-minute exercise sessions, to receive either a pedometer alone (control group); pedometer and information on its use; or pedometer, information, and rewards. Results showed that step count was greater amongst the group with rewards (mean 3,429; SD 458), compared to the pedometer plus information group (mean 2,091; SD 483) and control group (mean 2,655; SD 577) [19].

Another RCT study conducted among overweight men with a family history of T2DM in Saudi Arabia [18], had a 10,000 steps/day goal for the intervention group while the control group was asked to continue their normal PA level for 12 weeks. Results showed that compared to the control group, participants in the active group significantly increased their step count (~5000 steps/day) during the intervention programme (P<0.05). There were also changes within arms: the step count almost tripled among the intervention group at the end of the experimental period, while the control group had a 50% increase in step count compared to baseline.

A study performed in sedentary female university students in UAE [20] utilised a culturally-adapted 10 week PA programme with personalised step count goals (3000 additional steps per day from their baseline). There was no significant difference between step count at baseline for the intervention and control groups; after 10 weeks statistically significant increased step count were observed in the intervention group with no change in the control group (p = 0.001). Statistically significant changes in walking times were also observed across the groups (p = 0.01). However, the results of this RCT were not published in full due to losses to follow-up, weakening conclusions [20].

The remaining five interventions were part of a non-randomized community-based intervention in Qatar termed ‘Step into Health’ [25–27,29,30]. All five studies implemented the same intervention; goal-setting 10,000 steps/day using pedometers, and compared results to baseline values. One evaluation was performed only in women [29] whereas the study population in another was larger and 72.7% of participants were men [24]. All studies showed an increase in step count across the intervention period with daily step count, for example, increasing from 6,833 (SD 4,144) to 10,600 (SD 6,385) at week 12 in the study by Al-Kuwari et al [25].

#### Multi-component interventions including PA

The five multi-component intervention studies took place in a range of settings and three were targeted at clinically defined populations (i.e., focussing on adults with T2DM [23] and overweight adults [22]). The study by Taheri et al [22], focussed on overweight T2DM patients aiming at increasing step count to 10,000 per day. The results [22] showed an increased duration of walking in the intervention group which was reported in metabolic equivalent of task (MET) [35] minutes per week. In the intervention group, walking was significantly increased by 151.2 MET.min/week (SD 994.7) compared to a decrease in the control group of 235.7 MET.min/week (SD 652.0; 95% CI 160.3–660.3; P = 0.002). There was however no significant overall difference between the intervention and control group with regards to MET.min/week of moderate and vigorous exercise and total MET.min/week. Although PA was measured through the use of both accelerometers and the IPAQ, the more objective accelerometer results were not utilised in the study.

The study in Oman among T2DM primary care patients by Alghafri et al [23] performed an intensive intervention with face-to-face consultations at 0, 4 and 8 weeks and monthly motivational messages were sent via the smartphone application, WhatsApp. The study included accelerometers, pedometers and the GPAQ to measure PA. Less than half of the subjects (48% intervention vs 40% control) were given accelerometers and about 70% of all participants had data at baseline and at 12 months. The findings demonstrated significant between-group differences in favour of the intervention group for mean steps/day (+757, 95% CI 18 to 1) and sitting time hours/day (−1.5, 95% CI −2.4 to −0.7). Based on the GPAQ questionnaire at 12 months, the mean change in MET.min/week was significantly longer for the intervention group compared with the control group at +631.3 (95% CI 369.4 to 893.2) in the intervention group compared to +183.2 (95% CI 83.3 to 283.0) in the control group, with a between-difference of +447.4 (95% CI 150.7 to 744.1; P = 0.003).

Another study assessed a multi-component intervention undertaken in the workplace. Al-Mohannadi et al [28] conducted an NRI study using a subsample of the Qatar ‘Step into Health’ study population identified as hospital staff. Apart from providing a pedometer to staff members, health tips were regularly sent by email and message; and an internal ranking system for step count was also used to promote the 10,000 steps/day target. Results showed that the step count increased significantly from 7,890 to 9,270 during the intervention (P<0.05), though there was a slight but insignificant reduction during the five months post-intervention follow-up (8,998 steps).

The remaining two multi-component intervention studies were conducted among healthy individuals. Aside from introducing step-count pedometers [17], healthy Omani female participants were also asked to keep a food diary during the intervention. Al-Anqodi et al [17] found that both the intervention and control group increased their active time per day during the intervention period, though the increase was 33% greater in the intervention group (from 33min/day to 48 min/day) compared to the control group (from 26 min/day to 36 min/day). Kutbi et al [24] integrated a multi-component intervention technique including health education, group counselling and discussion on healthy lifestyle among male teenage students, but no significant difference in total METs was found between the intervention and control groups [24].

#### Effect of pedometer intervention on step count

**Fig 3:**
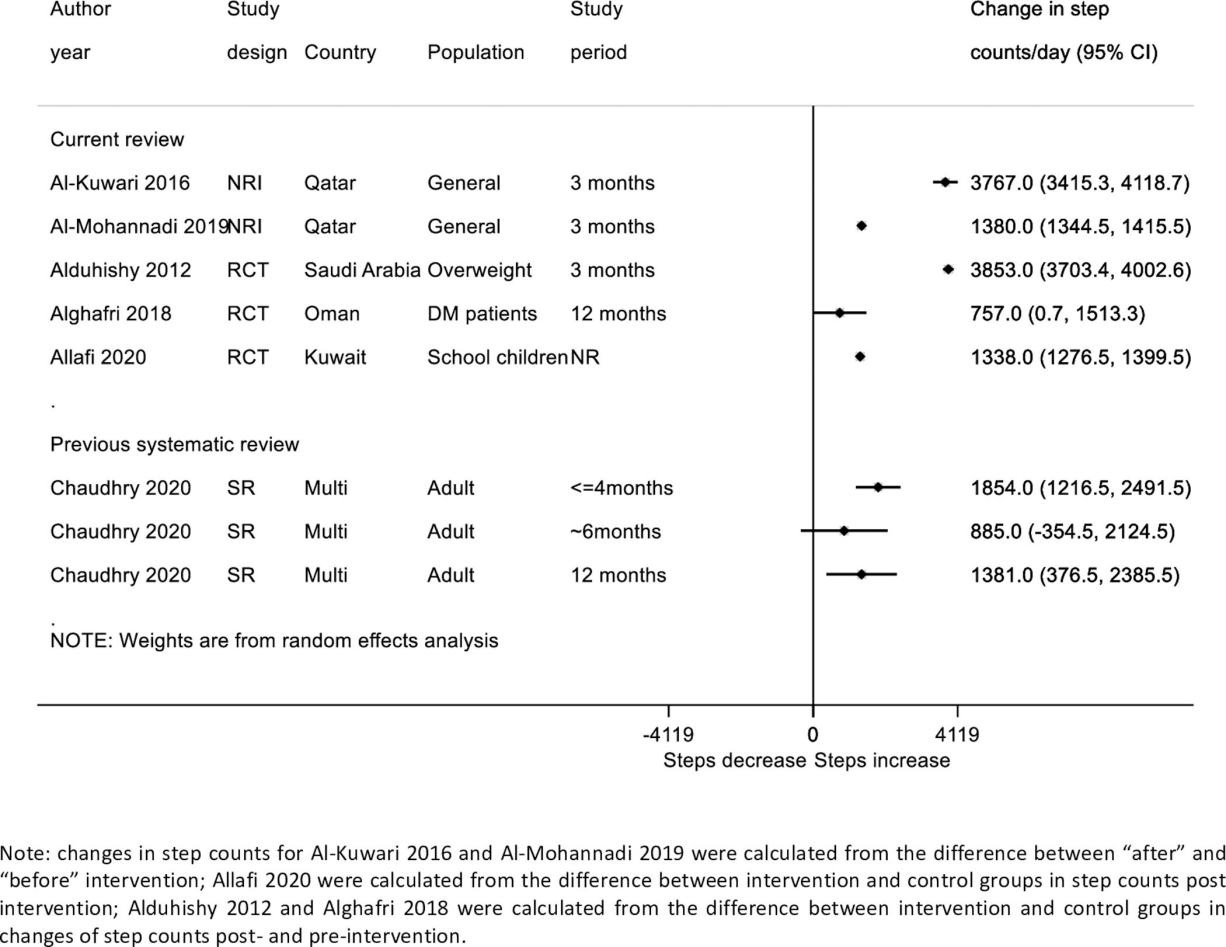
Forest plot of included randomized control studies on PA intervention on step count per day compared to summary estimates from previous systematic review by Chaudhry 2020.

Eleven of the included studies reported step count as an outcome; five of these were sub-studies from the “Step into Health” National program in Qatar. As it appeared the samples in these studies might be somewhat overlapping, we selected Al-Kuwari 2016 to represent the findings of these studies as this study reported changes in step count in sufficient detail to be estimated and plotted (see Fig 3) [25]. There was significant heterogeneity between the different studies reporting changes in step count. Large increases of approximately 3800 steps per/day were reported in two studies [18,25], whilst the smallest increase was found among T2DM patients in Oman (757 steps/day) [23].

## Discussion

### Key findings

This systematic review included 14 studies performed in six GCC countries to assess the effect of a variety of PA interventions on a range of both objective and subjective PA measures, and in some cases health-related secondary outcomes (particularly anthropometry and cardiovascular risk factors). Most of the included studies focussed on PA interventions alone, generally setting goals to increase step count. Five studies in total were identified that appeared to be part of the same study population; from the Qatari “Step into Health” programme, and it is possible that these were not mutually exclusive. Five of the randomized studies reported statistically significant increases in PA level compared to the control group, which would be of sufficient magnitude to have a health impact, if maintained over time. The five remaining studies included PA interventions as part of multi-component programmes with the majority providing additional health-related supports, such as advice on diet and glycaemic control. These were mostly targeted at people with or at high risk of developing T2DM, and PA changes were not the primary outcome and often not reported in detail.

All of the PA intervention-only studies utilised pedometers to measure step count. Two outlying studies showed particularly large increases in steps per day (around 3800 steps/day increase). One of these was the Al-Kuwari et al [25] “Step into Health” study from Qatar. The implementation of the study was not randomized and the study used volunteers with no external control group and reported results over a short time period (4 months). It is therefore possible that the substantial effect size was partly explained by selection biases. A very large benefit was also shown in the small study of 39 overweight men from Saudi Arabia [18]. Although there was substantial heterogeneity between studies, the other studies (the majority of which were randomized) showed more modest increases of approximately 500–1,000 steps/day; these findings are comparable to the level of change reported in systematic reviews internationally [16], and could be clinically important if they could be scaled up and sustained in Gulf populations [16] (see Fig 3).

Five studies that performed PA as part of a multi-component programme reported changes in some aspect of PA level post-intervention. Two of them reported a statistically significant increase in PA after intervention, while the others were either not statistically significant, or not clearly reported. A very tentative conclusion is that studies focussing primarily on PA, particularly through one modality such as step count (pedometers), may have larger effects on PA behaviours than multicomponent interventions, but this might be affected by the small sample size of most multi-component studies identified and potential biases in study design. This finding is not specific to the GCC setting and has been identified in other, larger global systematic reviews [16].

Nevertheless, PA interventions as part of a multi-component programme were found to improve anthropometric markers by lowering BMI, weight, waist-circumference, waist-to-hip ratio, fat mass, and BP as well as improving metabolic markers such as blood lipid levels (total cholesterol, high-density lipoproteins, low-density lipoproteins, triglycerides) and HbA1c. These markers are important for cardiovascular and metabolic health, and are also easier to measure objectively than PA. However, participants taking part in the multi-component interventions were mostly already obese, with pre-diabetes or T2DM, and the studies themselves were generally small and short term in nature. As such, they might be more motivated to try to change their lifestyle, or since their BMIs were higher it might be easier to lose weight and improve anthropometric outcomes. This mirrors international research of targeted intensive lifestyle change among people with pre-diabetes [36]; but uncertainty remains about the ability to scale-up such interventions to whole populations [37,38], particularly in the GCC countries.

### Strengths and limitations

The key strength of our review is the systematic search strategy performed, making it less likely to miss important regional studies. We identified and included several “grey literature” studies (e.g., PhD dissertations), not previously noted [5,11]. Whilst relatively few intervention studies were identified, most of those included had used at least some objective measure of PA (mostly pedometers). Two studies also had longer term follow-up (over five months) and seven included intervention or follow-up periods during the hottest time of the year (June–August), when it is hardest to maintain PA in the region.

The key limitation is that PA intervention studies in the region are still sparse. We also failed to obtain copies of two full texts we identified as potentially eligible despite repeated attempts. Included studies were heterogeneous with respect to i) study design ii) study population iii) primary objectives iv) assessment of outcomes v) duration of intervention and vi) follow-up period. This heterogeneity in addition to the relatively small number of studies made it difficult to perform any meta-analysis or sub-group analyses to explore differences between studies. A final limitation is the high levels of bias within studies. Only eight of the fourteen studies were RCTs and amongst them there were some significant limitations such as losses to follow-up and incomplete reporting of results. Several of the RCTs did not appear to have been analysed appropriately, reporting or focusing mainly on within group changes in the PA outcome rather than between group changes, and reported conclusions about statistical significance of any differences between groups could be misleading.

As some studies performed a multi-interventional programme, it is difficult to determine the extent to which reductions in anthropometric markers e.g., BMI and waist-circumference were due to the PA intervention as distinct from other components (such as dietary change). Further research will also be required to determine the relative effects of PA interventions alone–both quantitative, qualitative or a combination–when compared to those of dietary interventions alone or in combination with each other.

Furthermore, as only 14 studies were identified, some of which were from the same study population, we were unable to meet the secondary aims stated in our protocol [12]. These included analysing whether benefits were maintained beyond the end of the intervention period and performing subgroup analyses assessing the difference between PA uptake in men and women. Although several studies were performed solely among either men or women, only one presented results from men and women independently and only two studies included a period of follow up post-intervention. As such, further studies are required to demonstrate both the sustained effects of intervention programmes and the differential effects of these programmes on men and women in the GCC countries.

### Implications

Given the relatively limited number of intervention studies identified, further studies are clearly warranted. The preliminary evidence suggests that a focus on increasing PA uptake in whole populations using pedometers or other types of step counters might be most effective. Appropriate adaptions (e.g., early morning exercise programmes) at the coolest times of the day were reported by some studies. Whilst this was not investigated directly in the included studies, GCC countries could support flexible working arrangements that encourage breaks for PA and to break up sitting time, at the coolest times of day, or use of indoor air-conditioned spaces for exercise. PA levels are thought to be particularly low in women. Four of the studies were among women but further studies (at least with results disaggregated for men and women) are warranted to identify more clearly gender-specific barriers and facilitators. Only two RCTs took place in children; given the rising levels of obesity in children more research is clearly needed.

Another gap in this research area is that little inclusion exists for the totally inactive population (e.g., those with steps counts of 2000 or below at baseline). It may be easier to encourage somewhat active people (e.g., healthy volunteers, educated health professionals) to increase PA levels compared to those who are very inactive. However, the greatest health gain could arise in the most inactive groups [39].

Four published protocols were identified for studies that aimed to target PA in the Gulf region (S3 Appendix), some of which were either registered or implemented very recently without results yet. Two studies targeted female university students, and two targeted T2DM patients and overweight employees in a company. Though there seems to be more focus on female and unhealthy populations (i.e., those with T2DM), there is still lack of involvement of vulnerable populations who need more health attention; especially those who are less educated or unemployed. Nevertheless, the limited evidence supports the conclusions drawn from the wider body of evidence around PA interventions.

## Conclusion

Obesity is a rising problem in both adults and children in the GCC countries with these countries experiencing some of the highest levels in the world. Furthermore, the levels of PA in these countries are very low with societal, cultural and environmental factors contributing to a decreased uptake. There is a lack of studies performed in the area assessing the effect of PA interventions to improve the level of PA. However, the interventions with the greatest effect on PA in the GCC countries appear to be pedometer-based programmes implementing goal-setting, rewards-based systems and measuring step count. Other research on PA worldwide has also suggested that focussed, simple messages (e.g. to increase step count) may be most effective. The results of this review can be used by policy-makers to scale-up interventions to increase PA levels and health of the GCC citizens.

## Supporting information

S1 Checklist (DOC)

S1 Appendix Search strategy example in Medline. (PDF)

S2 Appendix Studies excluded during full text screening with reasons. (PDF)

S3 Appendix Study protocols identified during search (no research output found for these protocols). (PDF)

S4 Appendix Recommendations table. (PDF)
