# Supplementary material for: A systematic review of interventions to promote physical activity in six Gulf countries
Source: PLoS One. 2021 Oct 28;16(10):e0259058. doi: 10.1371/journal.pone.0259058 (PMC8553078; doi:10.1371/journal.pone.0259058)
Supplement: S1 Appendix — (PDF) [file pone.0259058.s002.pdf]

S1 Appendix. Search strategy example in Medline.

1. (exert*[tw]) OR (exercis*[tw]) OR (physical activi*[tw]) OR (physical fitn*[tw]) OR (sport*[tw]) OR (motor activ*[tw])

2. (walk*[tw]) OR (jog[tw]) OR (swim*[tw]) OR (cycl*[tw]) OR (run[tw]) OR (jogging[tw]) OR (running[tw])

3. (weight lift*[tw]) OR (strength train*[tw]) OR (resistance train*[tw]) OR (circuit train*[tw]) OR (weight train*[tw]) OR (aerobic train*[tw]) OR (cardio train*[tw]) OR (aerob*[tw]) OR (HIIT[tw]) OR (biking[tw])

4. "exercise"[MeSH Terms] OR "physical education and training"[MeSH Terms] OR "education"[MeSH Terms] OR "physical fitness"[MeSH Terms] OR "sports"[MeSH Terms] OR "physical exertion"[MeSH Terms] OR “motor activity”[MeSH Terms]

5. 1 OR 2 OR 3 OR 4

6. (randomized controlled trial[pt] OR controlled clinical trial[pt] OR randomized[tiab] OR randomised[tiab] OR placebo[tiab] OR clinical trials as topic[mesh:noexp] OR randomly[tiab] OR trial[ti] NOT (animals[mh] NOT humans [mh]))

7. (epidemiologic studies[MeSH Terms]) OR (case control studies[MeSH Terms]) OR (cohort studies[MeSH Terms]) OR (cross-sectional stud*[MeSH Terms]) OR (case control[tw]) OR (cohort stud*[tw]) OR (cohort analy*[tw]) OR (follow up stud*[tw]) OR (observational stud*[tw]) OR (longitudinal[tw]) OR (retrospective[tw]) OR (cross sectional[tw]) OR (follow-up[tw]) OR (followup[tw])

8. 6 OR 7

9. (GCC[tw]) OR (gulf cooperation council[tw]) OR (Gulf countr*[tw]) OR (Oil producing countr*[tw]) OR (United Arab Emirates[tw]) OR (uae[tw]) OR (oman[tw]) OR (bahrain[tw]) OR (qatar[tw]) OR (saudi arabia[tw]) OR (ksa[tw]) OR (kuwait[tw]) OR (gulf arabi*) OR (gulf persian) OR (arabian peninsula)

10. 5 AND 8 AND 9
