# Supplementary material for: A systematic review of interventions to promote physical activity in six Gulf countries
Source: PLoS One. 2021 Oct 28;16(10):e0259058. doi: 10.1371/journal.pone.0259058 (PMC8553078; doi:10.1371/journal.pone.0259058)
Supplement: S2 Appendix — (PDF) [file pone.0259058.s003.pdf]

S2 Appendix.

Reason 1: No primary outcomes (physical activity level) reported

1. Abdelbasset WK, Tantawy SA, Kamel DM, Alqahtani BA, Elnegamy TE, Soliman GS, Ibrahim AA: Effects of high-intensity interval and moderate-intensity continuous aerobic exercise on diabetic obese patients with nonalcoholic fatty liver disease: A comparative randomized controlled trial. *Medicine* 2020, 99(10):1-6
2. Alghafri TS, Al Harthi SM, Al-Ajmi F, Al-Farsi Y, Craigie AM, Bannerman E, Anderson AS: Acceptability of the "MOVEdiabetes" physical activity intervention in diabetes primary care settings in Oman: findings from participants and practitioners. BMC Public Health 2020, 20(1):1-10.
3. Al-Ghafri TS, Al-Harthi SM, Al-Farsi Y, Craigie AM, Bannerman E, Anderson AS: Perceived Changes in General Well-being: Findings from the 'MOVEdiabetes' Physical Activity Randomized Control Trial. Oman Medical Journal 2020, 35(4):44-55.
4. Alfawaz HA, Wani K, Alnaami AM, Al-Saleh Y, Aljohani NJ, Al-Attas OS, Alokail MS, Kumar S, Al-Daghri NM: Effects of Different Dietary and Lifestyle Modification Therapies on Metabolic Syndrome in Prediabetic Arab Patients: A 12-Month Longitudinal Study. Nutrients 2018, 10(3):383.
5. Amer OE, Sabico S, Alfawaz HA, Aljohani N, Hussain SD, Alnaami AM, Wani K, Al-Daghri NM: Reversal of Prediabetes in Saudi Adults: Results from an 18 Month Lifestyle Intervention. *Nutrients* 2020, 12(3):804.
6. Racil G, Coquart JB, Elmontassar W, Haddad M, Goebel R, Chaouachi A, Amri M, Chamari K: Greater effects of high- compared with moderate-intensity interval training on cardio-metabolic variables, blood leptin concentration and ratings of perceived exertion in obese adolescent females. *Biology of Sport* 2016, 33(2):145-152.
7. Said MA, Abdelmoneem M, Alibrahim MC, Elsebee MA, Kotb AAH: Effects of diet versus diet plus aerobic and resistance exercise on metabolic syndrome in obese young men. *Journal of Exercise Science & Fitness* 2020, 18(3):101-108.
8. Tomar R, Hamdan M, Al-Qahtani MH: Effect of low to moderate intensity walking and cycling on glycaemic and metabolic control in type 1 diabetes mellitus adolescent males: A randomized controlled trial. Isokinetics & Exercise Science 2014, 22(3):237-243.
9. Tomar RH, Hashim MH, Al-Qahtani MH: Effects of a 12-week aerobic training on glycemic control in type 2 diabetes mellitus male patients. Saudi Medical Journal 2013, 34(7):757-759.
10. Reversal of prediabetes in saudi adults: results from an 18 month lifestyle intervention. Nutrients 2020, 12(3).

11. Al Mulhim AA, Al Gazzar S, Al Bahnassy A, Ali MS: Effect of exercise program on the bone mineral density in sedentary females. *Bahrain medical bulletin* 2004, 26(2):42-44.
12. Alfawaz HA, Al-Daghri NM: Significant reduction in full metabolic syndrome in saudi subjects with elevated fasting glucose through an intensive lifestyle monitoring programme of 12 months. FASEB journal 2018, 32(1).
13. Alghamdi RQ: A randomized controlled trial of a 12-week intensive lifestyle intervention program at a primary care obesity clinic for adults in western Saudi Arabia. Saudi medical journal 2017, 38(8):837-845.
14. Alnasir FA, Masuadi EM: The effect of loss of body weight on lipid profile in overweight individuals. Saudi medical journal 2006, 27(5):687-692.
15. Nct: Lifestyle Intervention for Cardiovascular Disease Risk Factors Among Female Residents at the National Guard Residential City, Jeddah, Saudi Arabia.
16. Nct: A Lifestyle Intervention for Type 2 Diabetes Patients in Kuwait and Its Impact on Glycaemic Control. *https://clinicaltrialsgov/show/NCT03397225* 2018.
17. Adedeji OO, Oyakhire GK, Saeed AK, Ghamdi AI. Effectiveness of interventions to reduce coronary heart disease risk. West Afr J Med. 2011 May-Jun;30(3):197-201. PMID: 22120486
18. Abdelbasset WK: Resistance exercise versus aerobic exercise combined with metformin therapy in the treatment of type 2 diabetes: a 12-week comparative clinical study. Endocrine, metabolic & immune disorders drug targets 2020.
19. al-Muhailan AR, Ramadan J, Gjorgov AN, Moussa M: Exercise time relationship to coronary risk factors in Kuwaiti adult males 1985-1986. International journal of epidemiology 1989, 18(4):818-823.
20. Sadiya A, Abdi S, Abusnana S: Lifestyle Intervention for Weight Loss: a group-based program for Emiratis in Ajman, United Arab Emirates. Diabetes, metabolic syndrome and obesity : targets and therapy 2016, 9:101-108.
21. Seyam M, Kashoo F, Alqahtani M, Alzhrani M, Aldhafiri F, Ahmad M: Effect of Walking on Sand with Dietary Intervention in OverweightType 2 DiabetesMellitusPatients: A Randomized Controlled Trial. Healthcare (Basel, Switzerland) 2020, 8(4).

Reason 2: Study protocols

1. Alghafri TS, Alharthi SM, Al Farsi YM, Craigie AM, McLeod M, Anderson AS: Study protocol for "MOVEdiabetes": a trial to promote physical activity for adults with type 2 diabetes in primary health care in Oman. BMC Public Health 2017, 17(1):1-7.

2. Al-Ozairi E, Ridge K, Taghadom E, de Zoysa N, Tucker C, Stewart K, Stahl D, Ismail K: Diabetes and TelecommunicationS (DATES) study to support self-management for people with type 2 diabetes: a randomized controlled trial. BMC Public Health 2018, 18(1):1249-1249.
3. Isrctn: Health benefits of a COMmunity-based WALKing programme in Bahraini adults with type 2 diabetes. http://wwwwhoint/trialsearch/Trial2aspx?TrialID=ISRCTN49941929 2011.
4. Nct: Physical Activity Intervention to Promote Walking Among Female University Students. https://clinicaltrialsgov/show/NCT03143309 2017.
5. Nct: What'Sapp Based Intervention for Promoting Physical Activity Among Female College Students. https://clinicaltrialsgov/show/NCT04166955 2019.
6. Nct: A Workplace Exercise Intervention in Dubai to Improve Cardio-metabolic Health. https://clinicaltrialsgov/show/NCT04403789 2020.
7. Isrctn: An intervention examining the effect of an intensive lifestyle intervention consisting of a low energy diet and physical activity on weight loss in subjects with early type 2 diabetes.
8. Isrctn: The role of text messages in helping people to change to a healthy lifestyle to stop them from getting diabetes type 2. http://wwwwhoint/trialsearch/Trial2aspx?TrialID=ISRCTN10857643 2018.
9. Abd El-Kader SM, Al-Shreef FM, Al-Jiffri OH: Impact of aerobic exercise versus resisted exercise on endothelial activation markers and inflammatory cytokines among elderly. African health sciences 2019, 19(4):2874-2880.

Reason 3: Wrong population

1. Bhargava K: Aerobic plus resistance training was more effective than either alone for reducing HbA1c levels in type 2 diabetes. ACP Journal Club 2008, 148(2):36-36.
2. Lari H, Noroozi A, Tahmasebi R: Impact of Short Message Service (SMS) Education Based on a Health Promotion Model on the Physical Activity of Patients with Type II Diabetes. Malaysian Journal of Medical Sciences 2018, 25(3):67-77.
3. Effect of electronic education based on health promotion model on physical activity in diabetic patients. Diabetes and metabolic syndrome: clinical research and reviews 2017.
4. Chiao-Nan C, Lee-Ming C, Korivi M, Ying-Tai W: Home-Based Exercise May Not Decrease the Insulin Resistance in Individuals With Metabolic Syndrome. Journal of Physical Activity & Health 2015, 12(1):74-79.
5. Falkowski G, Knigge H, Montiel G, Wilke C, Pieper S, Wehner S, Weber K, Frobose I, Struder H, Predel HG: Effects of a 20-week stamina training on the physical performance and

anthropometric and metabolic parameters in older, overweight/obese, inactive men. Deutsche zeitschrift fur sportmedizin 2007, 58(7):279.

6. Ha AS, Burnett A, Sum R, Medic N, Ng JYY: Outcomes of the Rope Skipping 'STAR' Programme for Schoolchildren. Journal of Human Kinetics 2015, 45:235-242
7. Huong QN, Herting JR, Kohen R, Perry CK, LaCroix A, Adams-Campbell LL, Beresford SAA, Eaton CB, Tinker L: Recreational Physical Activity in Postmenopausal Women Is Stable Over 8 Years of Follow-Up. Journal of Physical Activity & Health 2013, 10(5):656-668.
8. Knigge H, Falkowski G, Montiel G, Wilke C, Sperlich B, Wehner S, Wienke C, Frobose I, Struder H, Predel HG: Effects of a 20-week stamina training (bicycle versus walking/running) on physical performance and systemic haemodynamic parameters in older, overweight/obese, inactive men. Deutsche zeitschrift fur sportmedizin 2007, 58(7):267.
9. Kozak A, Nguyen C, Yanos B, Fought A: Persuading Students to Exercise: What Is the Best Way to Frame Messages for Normal-Weight Versus Overweight/Obese University Students? *Journal of American College Health* 2013, 61(5):264-273.
10. Laque M, Becker S, Knigge H, Montiel G, Stronmann K, Predel HG: Different walking techniquies improve the cardiovascular risk profile in post-menopausal women with hypercholesterolaemia: postmenopausal exercise prevention programme (PEPP). Deutsche zeitschrift fur sportmedizin 2005, 56(7):207.
11. Leehey DJ, Collins E, Kramer HJ, Cooper C, Butler J, McBurney C, Jelinek C, Garabedian A, O'Connell S: Structured exercise in obese diabetic patients with chronic kidney disease: a randomized controlled trial (NCT01036490). American journal of kidney diseases 2016, 67(5):A67-.
12. Nct: Lifestyle Intervention for Prevention of Gestational Diabetes Mellitus in the UAE Population. https://clinicaltrialsgov/show/NCT04273412 2020.
13. Richter H, Kraft K, Weisser B: Increasing physical activity and reducing cardiovascular risk factors due to telephone intervention in type 2 diabetics. Deutsche zeitschrift fur sportmedizin 2007, 58(7):278.
14. Surakka J, Alanen E, Aunola S, Karppi S-L, Lehto P: Adherence to a Power-type Strength Training Programme in Sedentary, Middle-aged Men and Women. *Advances in Physiotherapy* 2004, 6(3):99-109.
15. Al Khalifah R, Thabane L, Tarnopolsky MA, Morrison KM. The prognosis for glycemic status among children and youth with obesity 2 years after entering a weight management program. Pediatr Diabetes. 2018 Aug;19(5):874-881. doi: 10.1111/pedi.12675. Epub 2018 Apr 17. Erratum in: Pediatr Diabetes. 2020 May;21(3):552. PMID: 29577539.

16. Abdelbasset WK, Tantawy SA, Kamel DM, Alqahtani BA, Soliman GS: A randomized controlled trial on the effectiveness of 8-week high-intensity interval exercise on intrahepatic triglycerides, visceral lipids, and health-related quality of life in diabetic obese patients with nonalcoholic fatty liver disease. Medicine 2019, 98(12):e14918.
17. Alansare A, Alford K, Lee S, Church T, Jung HC: The Effects of High-Intensity Interval Training vs. Moderate-Intensity Continuous Training on Heart Rate Variability in Physically Inactive Adults. International journal of environmental research and public health 2018, 15(7).
18. Lari H, Tahmasebi R, Noroozi A: Effect of electronic education based on health promotion model on physical activity in diabetic patients. Diabetes & metabolic syndrome 2018, 12(1):45-50.
19. Sanaeinasab H, Saffari M, Dashtaki MA, Pakpour AH, Karimi Zarchi A, O'Garo KN, Koenig HG: A Theory of Planned Behavior-Based Program to Increase Physical Activity in Overweight/Obese Military Personnel: A Randomised Controlled Trial. Applied psychology Health and well-being 2020, 12(1):101-124.

Reason 4: No sufficient information to be assessed

1. Al-Ajlan AR, Mehdi SR. Effects and a dose response relationship of physical activity to high density lipoprotein cholesterol and body mass index among Saudis. Saudi Med J. 2005 Jul;26(7):1107-11. PMID: 16047063.
2. Van der Walt M, Sayegh S, Al-Kuwari M. Effectiveness of a One-Month Pedometer-Based Intervention Challenge on Physical Activity Levels at the Workplace in Qatar: A Cohort Study. Journal of Aging and Physical Activity - Volume 24, Issue 0, pp. S110-S110.

Reason 5: No physical activity intervention

1. Armitage CJ, Alganem S, Norman P. Randomized Controlled Trial of a Volitional Help Sheet to Encourage Weight Loss in the Middle East. Prev Sci. 2017 Nov;18(8):976-983. doi: 10.1007/s11121-017-0807-z. PMID: 28643134; PMCID: PMC5636872.
2. Al-Arifi MN, Al-Omar HA: Impact of a multidisciplinary intensive education program on type 2 diabetes mellitus patients' glycemic control and cardiovascular risk factors. Saudi medical journal 2018, 39(7):705-710.
3. Reed RL, Revel AD, Carter AO, Saadi HF, Dunn EV: A controlled before-after trial of structured diabetes care in primary health centres in a newly developed country. International journal for quality in health care : journal of the International Society for Quality in Health Care 2005, 17(4):281-286.

Reason 6: Narrative review

1. Evans CE, Albar SA, Vargas-Garcia EJ, Xu F: School-Based Interventions to Reduce Obesity Risk in Children in High- and Middle-Income Countries. Advances in food and nutrition research 2015, 76:29-77.
2. Musaiger AO, Al Hazzaa HM, Al-Qahtani A, Elati J, Ramadan J, Aboulella NA, Mokhtar N, Kilani HA: Strategy to combat obesity and to promote physical activity in Arab countries. Diabetes, metabolic syndrome and obesity : targets and therapy 2011, 4:89-97.

Reason 7: No clear report of PA change

1. Al Saweer A, Salehi S, Al Tiho M, Alekri A, Al Hawaj H, Al Zayani S: Workplace health initiatives. Bahrain Medical Bulletin 2017, 39(4):216-219.
2. Mokabel FM, Aboulazm SF, Hassan HE, et al. The efficacy of a diabetic educational program and predictors of compliance of patients with noninsulin-dependent (type 2) diabetes mellitus in Al-Khobar, Saudi Arabia. J Family Community Med 2017;24(3):164-72
