# Supplementary material for: A systematic review of interventions to promote physical activity in six Gulf countries
Source: PLoS One. 2021 Oct 28;16(10):e0259058. doi: 10.1371/journal.pone.0259058 (PMC8553078; doi:10.1371/journal.pone.0259058)
Supplement: S3 Appendix — (PDF) [file pone.0259058.s004.pdf]

S3 Appendix.

Trial title	Year of registration	Country	Population	Study design	Intervention and control	Duration of intervention	Outcome measures
Health benefits of a COMmunity-based WALKing programme in Bahraini adults with type 2 diabetes	2011	Bahrain	Adults aged 24-60 with type 2 diabetes	RCT	Standard diabetes treatment alone; standard diabetes treatment with physical activity education to promote walking; or standard treatment with physical activity education to promote walking and a step counter to record daily walking activity.		Blood glucose, physical activity level, fitness and body composition assessed at the start, middle (6 weeks) and end (12 weeks) of the study
Physical Activity Intervention to Promote Walking Among Female University Students	2017	Saudi Arabia	18-28 years old female university students	RCT	Experimental: Walking + WhatsApp<br><br>The participants assigned to the intervention group will be given a brief (15-minute) orientation where they learn about the importance of exercise, diet, and the benefits of weight reduction. They will be given further instruction on the regular use of the pedometer. They will be enrolled into a WhatsApp group. They will receive 2-3 health-promotional (walking and diet) messages per week via WhatsApp.<br><br>Active Comparator: WhatsApp Only<br><br>The control participants will be enrolled into a WhatsApp group. They will receive 2-3 non-health related messages per week via WhatsApp.		Changes in walking (measured by pedometer); self-reported walking; BMI; barriers to weight management
What'Sapp Based Intervention for Promoting Physical Activity Among Female College Students	2019	Saudi Arabia	18-28 years old female university students	RCT	Experimental: (Intervention)<br><br>Participants will be provided with weekly messages including information for promoting physical activity.<br><br>No Intervention: (Control)	10 weeks	Changes in self-reported physical activity

					Participants will be evaluated without providing any intervention.		
A Workplace Exercise Intervention in Dubai to Improve Cardio-metabolic Health	2020	UAE	18-59 years central obese employee in the company	RCT	Experimental: Intervention Group<br><br>During the intervention period they will receive 2 hours per week of group exercise sessions during working hours for 12 weeks. However, after the intervention period, there will be no change in working hours<br><br>Active Comparator: Delayed Intervention Group (Control group)<br><br>During the intervention period there will be no change in working hours. However, after the intervention period they will receive 2 hours of exercise time during working hours per week for 4 weeks	12 weeks	Changes in high blood pressure, waist circumference, LDL cholesterol, HbA1c, fasting plasma glucose, prevalence of cardiovascular risk factors, BMI, self-reported and objectively measured physical activity level (with a further follow up of 4 weeks after the intervention)

Abbreviation: RCT- randomised control trial; UAE-United Arab Emirates
