# Supplementary material for: A systematic review of interventions to promote physical activity in six Gulf countries
Source: PLoS One. 2021 Oct 28;16(10):e0259058. doi: 10.1371/journal.pone.0259058 (PMC8553078; doi:10.1371/journal.pone.0259058)
Supplement: S4 Appendix — (PDF) [file pone.0259058.s005.pdf]

S4 Appendix

Implications	Recommendations
• Limited number of intervention studies identified	• Increasing PA uptake may be most effective using goal setting monitored by pedometers
• Further studies required in women to identify gender-specific barriers and facilitators of PA	• Appropriate adaptations required for this region – GCC countries should support flexible working arrangements that encourage breaks for PA at the coolest times of the day or use of indoor air-conditioned spaces
• Further studies required in children due to rising levels of obesity in this population (only 2 identified)	
• Further studies required in the truly inactive population – step count studies included individuals with “reasonable” step counts at baseline	
• Further studies required in those who need more health attention e.g. less educated or employed as they are less likely to volunteer for an intervention programme	
